# Prevalence of coeliac disease in patients with systemic lupus erythematosus: a systematic review and meta-analysis

**DOI:** 10.1136/lupus-2023-001106

**Published:** 2024-02-13

**Authors:** Adonis Sotoodeh, Madeleine Nguyen Hoang, Karin Hellgren, Anders Forss

**Affiliations:** 1 Medical Epidemiology and Biostatistics, Karolinska Institutet, Stockholm, Sweden; 2 Gastroenterology Unit, Department of Gastroenterology, Dermatovenereology and Rheumatology, Karolinska University Hospital, Stockholm, Sweden; 3 Department of Medicine, Solna, Division of Clinical Epidemiology, Karolinska Institutet, Stockholm, Sweden

**Keywords:** Antibodies, Autoimmune Diseases, Lupus Erythematosus, Systemic, Prevalence, Epidemiology

## Abstract

**Background:**

There is some evidence of a higher prevalence of coeliac disease (CD) among patients with SLE than in the general population. However, the prevalence estimates vary substantially.

**Objective:**

To investigate the prevalence of CD among patients with SLE through systematic review and meta-analysis.

**Methods:**

We performed searches in the databases of Medline, Embase, Cochrane and Web of Science Core Collection between 1 January 1990 and 9 July 2023. A total of 2053 publications were rendered in the searches, of which 68 were reviewed in full text and 14 included in the analyses. Primary analysis estimated the pooled prevalence of biopsy-verified CD in patients with SLE. In the secondary analysis, the prevalence of serological markers indicative of CD was investigated. The quality of studies was appraised using the Joanna Briggs Institute Critical Appraisal Tool. We conducted meta-regression analyses to investigate associations between the prevalence of CD in individuals with SLE and publication year, study population size, CD prevalence in the general population, proportion of females and quality assessment score.

**Results:**

A total of 14 studies met the inclusion criteria, of which 11 were included in the primary analysis of biopsy-verified CD. Among 1238 patients with SLE, 14 had CD. The weighted pooled prevalence of CD was 0.7% (95% CI 0.0 to 1.8). The weighted pooled prevalence of CD serological markers in 1063 patients with SLE was 3.7% (95% CI 1.4 to 6.7). In meta-regression analyses, no associations between CD prevalence and study characteristics, demographics and quality assessment scores were found.

**Conclusions:**

In this meta-analysis, we found a weighted pooled prevalence of biopsy-verified CD in patients with SLE comparable with the prevalence in the general population. Our findings do not support routine screening for CD in patients with SLE. However, individual screening could be considered in cases of clinical suspicion and additional risk factors for CD.

**PROSPERO registration number:**

CRD42022339594.

**WHAT IS ALREADY KNOWN ON THIS TOPIC**

- SLE has been linked to an increased risk of developing several other autoimmune diseases, including coeliac disease.

**WHAT THIS STUDY ADDS**

- In this meta-analysis, the weighted pooled prevalence of biopsy-verified coeliac disease in patients with SLE was similar to that of the general population.

**HOW THIS STUDY MIGHT AFFECT RESEARCH, PRACTICE OR POLICY**

- Our findings do not support the implementation of routine screening for coeliac disease in patients with SLE.
- Individual screening could be considered in cases of clinical suspicion or additional risk factors for coeliac disease.

## Introduction

SLE is the prototype of systemic autoimmune disease affecting multiple organ systems, with higher prevalence among females and with a peak onset between 15 and 35 years of age.^1^ Cutaneous, cardiopulmonary, joint and renal manifestations are the most common presentations, whereas gastrointestinal manifestations are comparatively more rare.^2^ The global prevalence is estimated to be around 0.1%.^3^ Although potential triggers of the disease continue to be discovered, the aetiology is still unclear.^4 5^ The risk factors for SLE consist of both genetic and environmental factors, including ultraviolet light exposure, hormonal factors, some infections and adverse effects from certain DNA-altering medications.^4^ SLE is a severely disabling condition and patients often experience a considerably reduced health-related quality of life.^6^ It is also associated with an increased mortality.^6^ Disease associations have been implied with several other autoimmune conditions such as Sjögren’s syndrome, antiphospholipid syndrome, autoimmune thyroid disease and coeliac disease (CD).^7–10^

CD is an autoimmune condition of the intestines characterised by gluten intolerance, believed to be caused by factors related to genetic predisposition and environmental exposures.^10^ Some of the most well-defined factors contributing to genetic predisposition for CD are the human leucocyte antigen (HLA) genes HLA-DQ2 and HLA-DQ8, which are responsible for antigen presentation to immune cells.^11^ The genes do not confer an absolute disease risk; however, in the absence of both HLA-DQ2 and HLA-DQ8, it is rare to develop CD.^11^ Other aetiological factors include environmental triggers and a dysregulated immune system.^12^ Apart from gluten exposure, possible associations with infections and dietary patterns have been proposed.^12^ Immunologically, an increased mucosal permeability from a compromised epithelial barrier enables an interaction between gliadin and T lymphocytes, followed by a cascading immune response.^12^ Approximately 0.5%–2.0% of the global population is affected by CD.^13^ A systematic review of CD by Singh *et al*^14^ presented a pooled prevalence of biopsy-verified CD with regional variances, with the highest prevalence in Europe and Oceania (both 0.8%), followed by Asia (0.6%), North America and Africa (both 0.5%), and the lowest in South America (0.4%). CD was also more prevalent in the paediatric population (0.9%) compared with the global adult population (0.5%).^14^ Similar to SLE, more females than males are diagnosed with CD.^15^ Symptoms of CD include malnutrition, chronic diarrhoea, poor weight gain and abdominal pain; however, a sizeable proportion of patients with CD are asymptomatic.^16^ There are additionally several described extraintestinal manifestations of CD, with common presentations including anaemia, arthralgia, dermatitis herpetiformis, osteoporosis and neuropathy.^17 18^ While adults may present with diarrhoea and anaemia, children are more likely to exhibit the hallmark signs of CD, including failure to thrive, abdominal distention and malabsorption.^19^

To our knowledge, no systematic review and meta-analysis has investigated the pooled prevalence of CD in patients with SLE. We therefore aimed to estimate this prevalence through a systematic review and meta-analysis of existing literature.

## Methods

This systematic review and meta-analysis was reported according to the Preferred Reporting Items for Systematic Reviews and Meta-Analyses guidelines.^20^ A study protocol was registered in the PROSPERO database (protocol ID: CRD42022339594).

### Search strategy

We searched the databases of Medline, Embase, Cochrane and Web of Science Core Collection from 1 January 1990 to 9 July 2023. Equivalent search strategies were applied for all databases (complete search strategies are presented in online supplemental figure S1). Only publications in the English language were included. Two reviewers, AS and MNH, independently screened the search results. Disagreement on inclusion of studies and interpretation of data was resolved by discussion between the reviewers, and in unresolved cases through mediation by AF. The reference lists of all publications eligible for full-text screening were screened for additional publications not captured by the search strategies.

10.1136/lupus-2023-001106.supp1 Supplementary data

### Identification of patients

#### Coeliac disease

For the diagnosis of CD, we required a reported small intestinal biopsy with Marsh stage II or III. The original Marsh classification system ranges from Marsh stage 0 to stage IV, defined as preinfiltrative (0), infiltrative (I), hyperplastic (II), destructive (III) and irreversible hypoplastic/atrophic lesions (IV).^21^ However, a modified Marsh classification system which ranges from stage 0 to stage IIIc was developed for diagnostic purposes to distinguish between the extent of villous atrophy (mild, marked and total) and is also widely used as a histopathological classification system for CD.^22^ In studies where CD was reported as ‘biopsy-verified CD’ or alike, the biopsy was presumed to be Marsh stage II or III. Study participants with reported Marsh stage I were not classified as patients with CD since previous studies in the field suggest that only relying on a stage I biopsy might overestimate the prevalence of CD. In the secondary analysis, we also investigated the prevalence of positive CD serological markers (antitissue transglutaminase antibodies (TTG) IgA/IgG and antiendomysial antibodies (EMA) IgA/IgG). In additional subanalysis, we analysed the prevalence of positive CD serological markers with the addition of antigliadin antibodies (AGA) IgA/IgG, which has a lower sensitivity and specificity for CD.^23^

#### Systemic lupus erythematosus

For SLE diagnosis, we required information in the studies that international diagnostic classification criteria for SLE, either the American College of Rheumatology,^24–26^ the Systemic Lupus International Collaborating Clinics^27^ or the European Alliance of Associations for Rheumatology,^26^ were applied. We also included studies where patients had their diagnosis of SLE assigned at specialised rheumatology clinics. Participants of all ages were included, both children and adults. The study characteristics are presented in table 1.

**Table 1:** Studies included in the meta-analysis of CD in patients with SLE

Study	Country	Prevalence of CD in the general population (%)	Patients (n)	Female (%)	Age mean (range)	Biopsy-verified CD, n (%)	Biopsied* (n)	Villous atrophy†	Antibodies*	Seropositive, n (%)	Diagnostic criteria
Biopsy-verified CD (n=11)											
Aikawa *et al*^95^	Brazil	0.4	41	85.4	14 (NA)	1 (2.4)	1	Yes	EMA	1 (2.4)	ACR
AlEnzi *et al*^96^	Saudi Arabia	1.3	115	90.4	27 (NA)	0 (0.0)	13‡	Unspecified	TTG/EMA/AGA	15 (13.0)	SLICC
Ben Abdelghani *et al*^97^	Tunisia	0.2	24	91.7	36 (18–52)	1 (4.2)	24	Unspecified	TTG/EMA/AGA	7 (29.2)	ACR
Elhami *et al*^98^	Iran	0.4	100	92.0	49 (16–76)	1 (1.0)	1	Yes (Marsh III)	TTG	1 (1.0)	ACR
Linzmeyer *et al*^99^	Brazil	0.4	281	93.2	NA (NA)	1 (0.4)	NA	Unspecified	–	–	ACR
Marai *et al*^100^	Italy	0.6	100	88.0	31 (15–56)	1 (1.0)	1	Yes (Marsh III)	TTG/EMA	3 (3.0)	ACR
Picceli *et al*^101^	Brazil	0.4	194	92.8	39 (17–67)	0 (0.0)	11	Unspecified	TTG/EMA	11 (5.7)	ACR
Rensch *et al*^94^	USA	0.7	103	NA	NA (NA)	0 (0.0)	24	Unspecified	EMA/AGA	24 (23.3)	Clinical
Şahin *et al*^102^	Turkey	0.9	50	88.0	15 (NA)	0 (0.0)	0	Unspecified	TTG/EMA	3 (6.0)	ACR
Shamseya *et al*^103^	Egypt	1.0	100	90.0	35 (19–55)	6 (6.0)	10	Yes (Marsh III)	TTG/EMA	10 (10.0)	SLICC
Soltani *et al*^93^	Iran	0.4	130	81.5	32 (16–60)	3 (2.3)§	49	Yes (Marsh II–III)	TTG/EMA/AGA	6 (4.6)¶	ACR
Solely CD serology (n=3)											
Caio *et al*^105^	Italy	0.6	35	82.9	NA (NA)	–	–	–	TTG/EMA/AGA	3 (8.6)	ACR
Gheita *et al*^104^	Egypt	1.0	10	80.0	12 (NA)	–	–	–	TTG	6 (60.0)	Clinical
Mader *et al*^106^	Israel	0.6	61	NA	NA (NA)	–	–	–	EMA/AGA	27 (44.3)	ACR

*‘–’: not studied; ‘0’:no cases of biopsy-verified CD.

†‘Yes’: when it was stated that biopsy-verified cases showed villous atrophy (and in some cases Marsh stage II or III).

‡Two patients with seropositive CD markers declined biopsy, either from the SLE group or the control group. At least 13 patients were biopsied.

§Three patients had biopsy-verified CD, of whom one was reported with Marsh stage III and two with Marsh stage II.

¶Two patients in the non-CD group tested positive for AGA and one patient tested positive for EMA. Overlap for seropositivity in the non-CD group is not reported. At least six patients in total tested positive for any of the CD serological markers.

ACR, American College of Rheumatology; AGA, antigliadin antibodies; CD, coeliac disease; EMA, antiendomysial antibodies; NA, data not available; SLICC, Systemic Lupus International Collaborating Clinics; TTG, antitissue transglutaminase antibodies.

### Data items and risk of bias

Data on the following items were retrieved: (1) first author and year of publication, (2) country and region, (3) study population size, (4) proportion of females, (5) Marsh classification, and (6) serological markers of CD (table 1). Because the prevalence of CD in the general population varies globally, we also investigated the prevalence of CD in SLE in relation to the prevalence in each country (table 1) (Brazil,^28^ Egypt,^29^ Iran,^30^ Italy,^31^ Israel,^32^ Saudi Arabia,^33^ Tunisia,^34^ Turkey^35^ and USA^36^). The Joanna Briggs Institute Critical Appraisal Tool was applied to evaluate the quality of the included publications (online supplemental table S2).^37^ Possible publication bias was assessed by funnel plot and Egger’s test.

### Statistics

We used the metaprop statistical command in STATA to conduct the meta-analyses.^38^ Metaprop is based on the STATA ‘metan’ command, which is used to pool estimates.^38^ The metaprop command also implements specific measures using binomial distribution to model the variability within studies or the Freeman-Tukey double arcsine transformation to stabilise variances, and is therefore expedient for conducting analyses with proportions close to or at the limits (0.0% and 100.0%).^38^ Heterogeneity between studies was analysed using Cochran’s Q test and denoted as I^2^. We defined I^2^ >50.0% as substantial heterogeneity. We anticipated a large heterogeneity among the included studies and consequently used a random effects model to estimate the weighted pooled prevalence. Statistical significance was set at p value <0.05.

To evaluate the robustness of our results, we conducted subgroup analyses stratified by study population size (<100 vs ≥100), Marsh stage III (yes/no) and region (Asia: Iran, Israel and Saudi Arabia; Europe: Italy and Turkey; North Africa: Egypt and Tunisia; North America: USA; South America: Brazil). We also performed meta-regression analyses to investigate associations between the prevalence of CD in individuals with SLE and publication year, study population size, CD prevalence in the general population, proportion of females and quality assessment score of the studies. These variables were chosen because they were presumed to potentially have an impact on the prevalence of CD when comparing studies. The meta-regression analyses were restricted to studies investigating the prevalence of biopsy-verified CD.

All analyses were performed using STATA V.17. Data extraction and compilation was conducted using Microsoft Excel (V.16.77.1, 2023; Microsoft, USA).

### Patient and public involvement

Patients and the public were not involved in the design, conduct, reporting or dissemination of this research.

## Results

In this study, titles and abstracts of 2053 publications were screened (online supplemental figure S1). Of these, 68 publications were eligible for a full-text review. No additional publications were added after screening the reference lists of these publications. A total of 54 publications were excluded. The reasons for exclusion were as follows: insufficient definition of outcome or exposure (n=48),^39–86^ overlapping study populations (n=3)^87–89^ and not in English language (n=3).^90–92^ The remaining 14 included publications (11 in the primary analysis and additionally 3 in the secondary analysis) comprised a total of 1344 patients with SLE (table 1).

### Prevalence of CD in SLE

A total of 11 articles fulfilled the inclusion criteria for biopsy-verified CD diagnosis and were included in the primary analysis.^93–103^ The mean study population for the included studies was 113 (range 24–281), with eight studies having a study population of ≥100 patients with SLE (table 1). Seven studies reported biopsy-verified cases of CD with prevalence estimates between 0.4% and 6.0%.^93 95 97–100 103^ The remaining four studies reported no cases of CD (study populations: n=115, n=194, n=103 and n=50).^94 96 101 102^ Among a total of 1238 patients with SLE included in the primary analysis, CD was found in 14 patients, rendering an unweighted prevalence of 1.1% (table 1). Four studies (n=430) reported Marsh stage III biopsy-verified CD cases (n=9), yielding an unweighted prevalence of 2.1%.^93 98 100 103^

**Figure 1:**
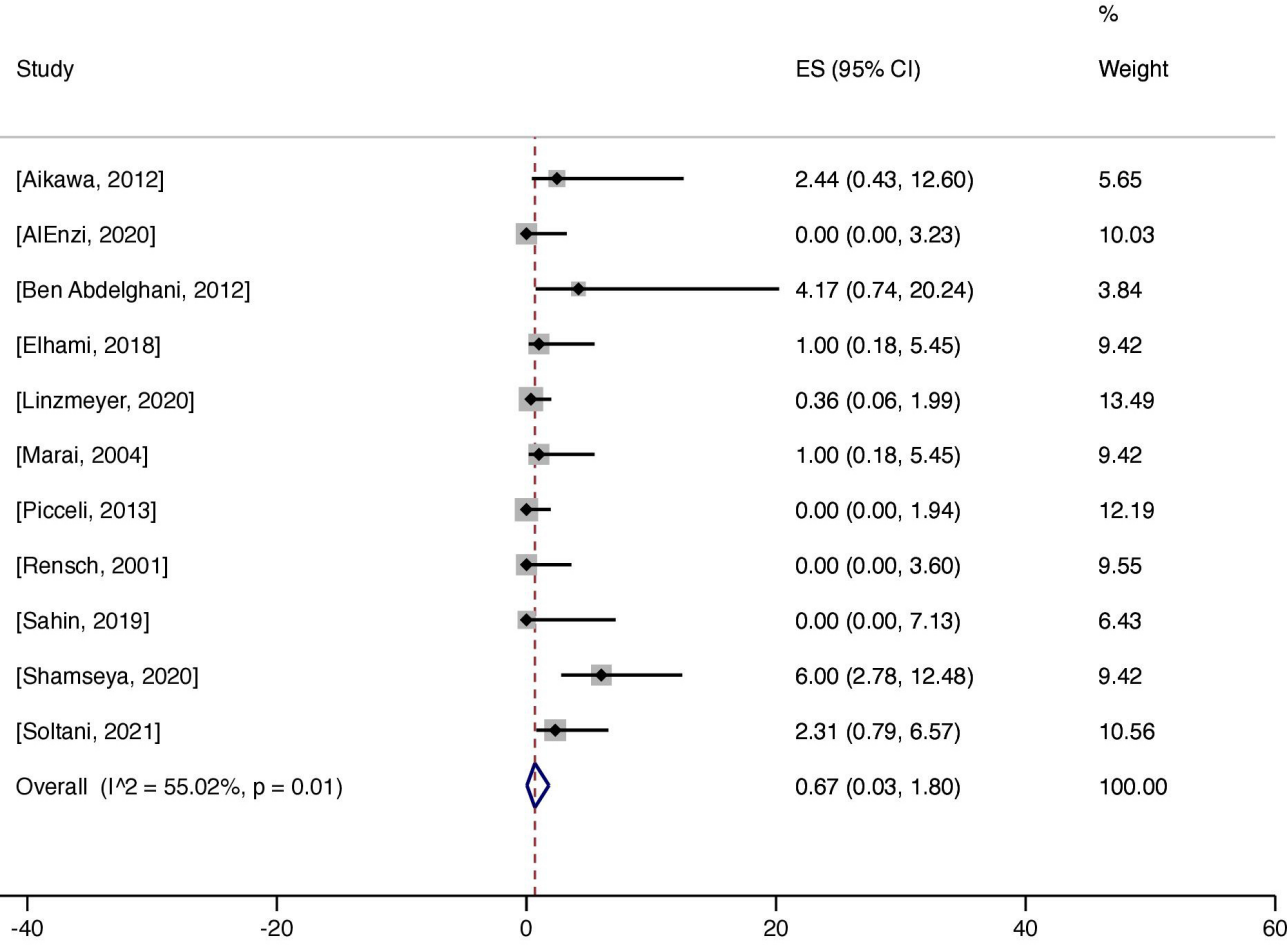
Prevalence of biopsy-verified coeliac disease in patients with SLE. ES denotes effect size. I^2^ indicates heterogeneity.

**Figure 2:**
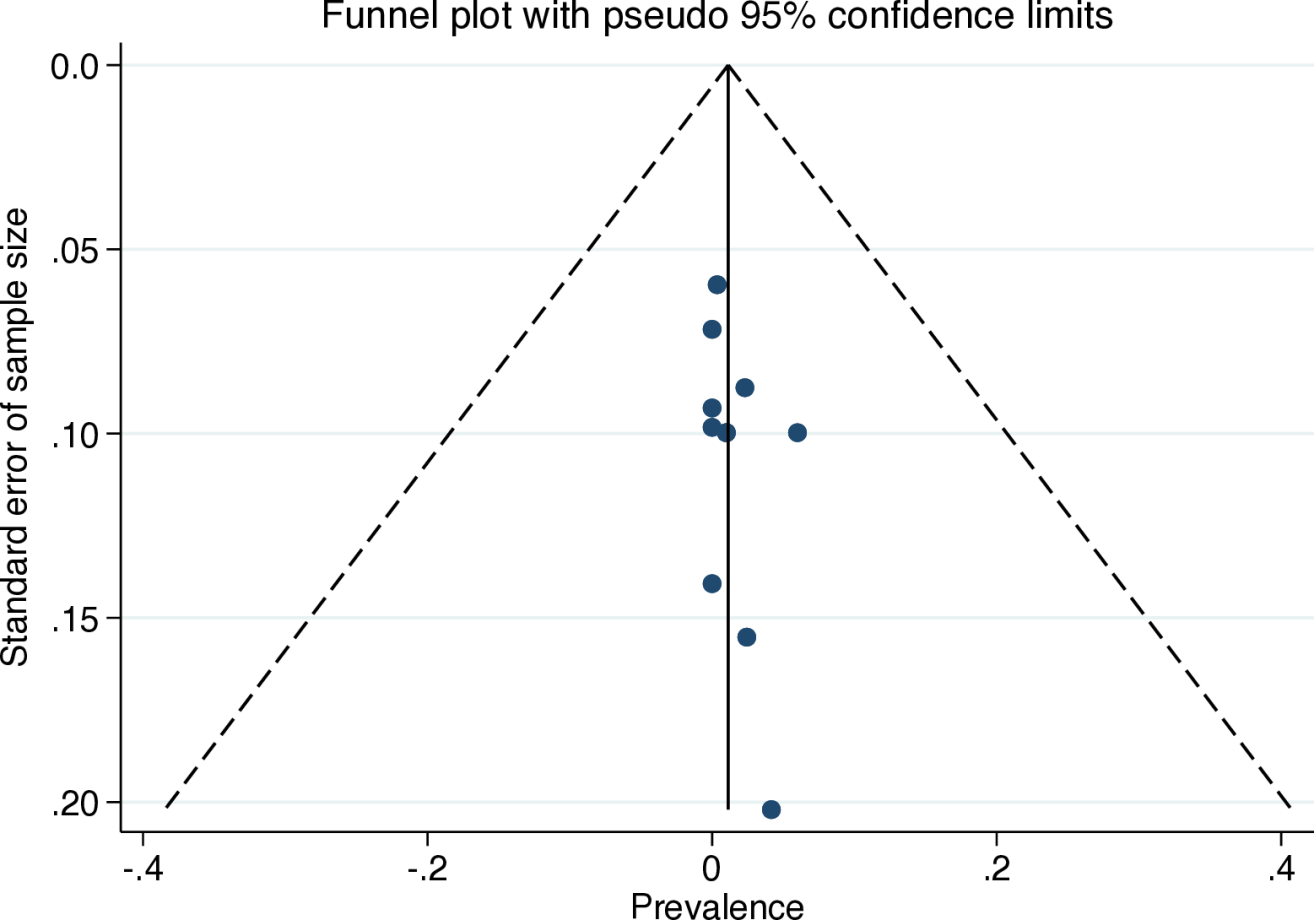
Funnel plot of studies investigating biopsy-verified coeliac disease in patients with SLE.

The weighted pooled prevalence of biopsy-verified CD was 0.7% (95% CI 0.0 to 1.8) (figure 1). The heterogeneity between studies was substantial (I^2^=55.0%, p=0.01). Stratified by study population size <100 (n=3) vs ≥100 (n=8),^93–103^ the weighted pooled prevalence was 1.1% (95% CI 0.0 to 5.0) compared with 0.7% (95% CI 0.0 to 2.0). Restricted to studies reporting Marsh stage III biopsy-verified CD, the estimated weighted pooled prevalence of Marsh stage III cases was 1.8% (95% CI 0.3 to 4.2) (online supplemental figure S2). In comparison by region, North Africa (n=2) showed the highest weighted pooled estimate (5.2%; 95% CI 1.7 to 10.2).^97 103^ Asia (n=3) had the second highest (0.8%; 95% CI 0.0 to 2.8),^93 96 98^ followed by Europe (n=2) (0.5%; 95% CI 0.0 to 2.7)^100 102^ and South America (n=3) (0.1%; 95% CI 0.0 to 1.2)^95 99 101^ (online supplemental figure S3). North America was represented by one study which reported no case of biopsy-verified CD.^94^ Meta-regression analyses of the association between CD prevalence in patients with SLE and publication year (p=0.58), study population size (p=0.26), prevalence of CD in the general population (p=0.78), proportion of females (p=0.66) and quality assessment score (p=0.84) showed no significant associations (online supplemental figure S4A–E). We found no significant publication bias in the funnel plot (figure 2) and Egger’s test (p=0.25).

### Prevalence of serological markers of CD in patients with SLE

**Figure 3:**
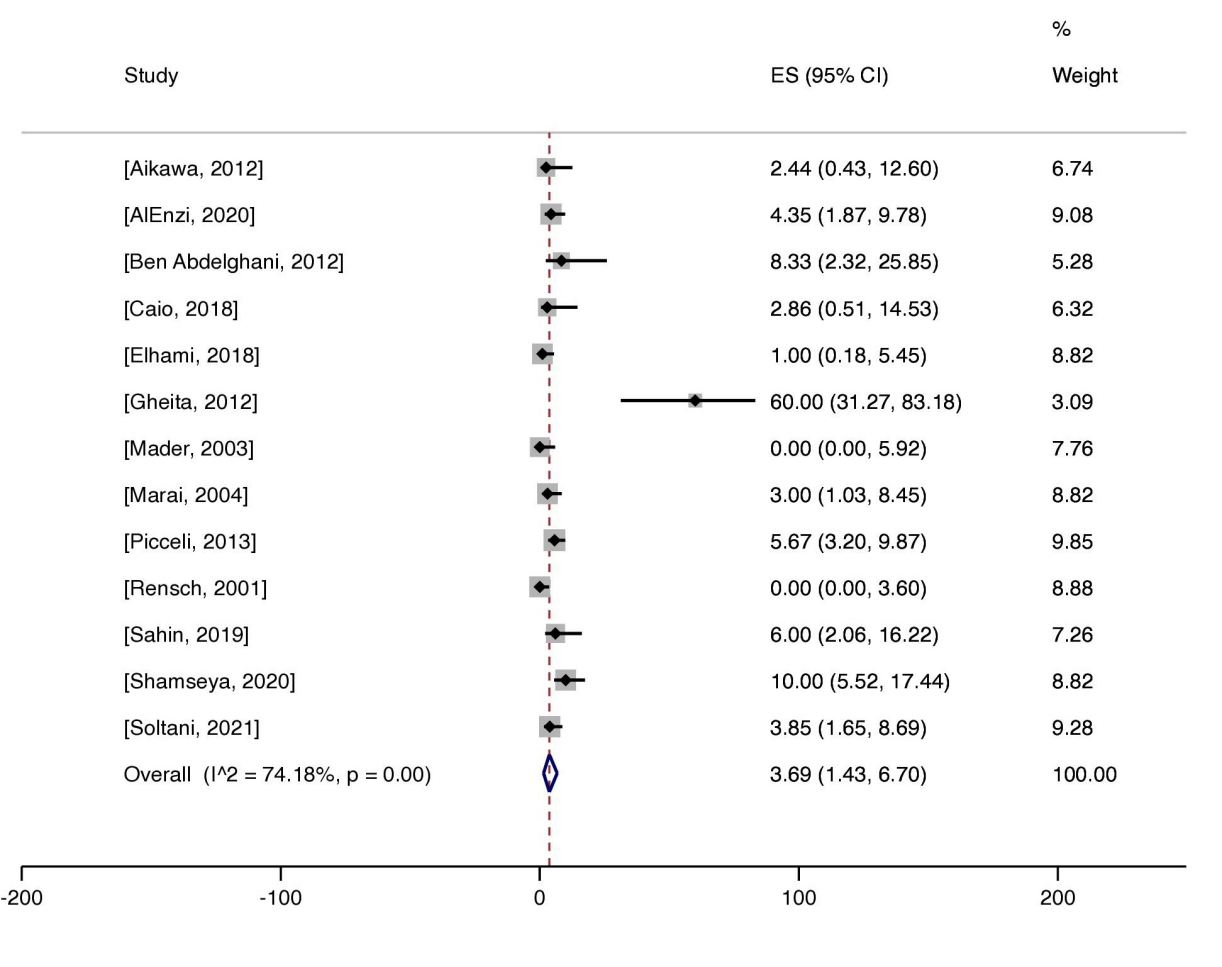
Prevalence of coeliac disease antibodies (antitissue transglutaminase and antiendomysial antibodies) in patients with SLE. ES denotes effect size. I^2^ indicates heterogeneity.

The prevalence of antibodies indicative of CD (TTG and EMA) was studied in the secondary analysis of 13 publications of this meta-analysis.^93–98 100–106^ One study did not report the prevalence of positive serological markers and was therefore not included in the analysis.^99^ Out of 1063 patients with SLE included in the secondary analysis, 4.5% (n=48) were positive for any of the serological markers TTG and EMA (online supplemental table S1). The weighted pooled prevalence was 3.7% (95% CI 1.4 to 6.7, I^2^=74.2%, p<0.01) (figure 3). One study reported a prevalence of >20.0%.^104^ When excluding that study, the weighted pooled prevalence was 3.1% (95% CI 1.4 to 5.2, I^2^=59.6%, p<0.01). With a broader definition of CD seropositivity, including a positive test for any of TTG, EMA and AGA, the pooled prevalence was 11.6% (95% CI 5.9 to 18.7, I^2^=89.3%, p<0.01) (online supplemental figure S5). When excluding studies which reported a prevalence of >20.0% (n=4),^94 97 104 106^ the weighted pooled prevalence was estimated to be 5.5% (95% CI 3.1 to 8.4, I^2^=59.2%, p=0.01).

## Discussion

This systematic review and meta-analysis investigated the prevalence of biopsy-verified CD in patients with SLE along with the association between CD prevalence and demographic and study-related factors. In a total of 1238 patients with SLE, we found a pooled weighted prevalence of biopsy-verified CD of 0.7%. This is equal to 1 in 149 patients with SLE diagnosed with CD. To our knowledge, this is the first meta-analysis to investigate the prevalence of CD in patients with SLE.

We report a weighted pooled prevalence of 0.7% for biopsy-verified CD in patients with SLE that is similar to the global prevalence of CD. The prevalence of biopsy-verified CD in the 11 included studies ranged from 0.0% to 6.0%. The reported prevalence of CD in patients with other autoimmune diseases varies substantially. A recent meta-analysis reported a pooled prevalence of 0.4% (95% CI 0.0 to 1.2) in patients with rheumatoid arthritis and 1.4% (95% CI 0.7 to 2.2) in patients with juvenile idiopathic arthritis.^107^ Another meta-analysis of CD in patients with autoimmune thyroid disease showed a pooled prevalence of 1.6% (95% CI 1.3 to 1.9).^108^ The differences in the reported prevalence suggest that immunopathogenic factors associated with CD may vary by type of autoimmune disease.

The underlying mechanisms associating CD with SLE are likely multifactorial and are not fully understood. The HLA risk genes HLA-B8 and HLA-DR3 are shared between CD and SLE^109^ and may play a role, independently or synergistically, in the pathophysiology of both diseases. The HLA-DR3 haplotype is often present together with the DQ2 allele, which is seen in the vast majority of individuals with CD. Moreover, it is estimated that two out of three individuals with SLE carry the HLA-DR2 or HLA-DR3 haplotypes.^110^

Moreover, activation of the innate immune system through toll-like receptors (TLRs) and subsequent production of interferons could indicate a potential common pathophysiological mechanism for CD and SLE. In a recent review by Talipova *et al*^111^, TLRs were introduced as possible genetic factors in the aetiology of CD due to their potential role in the interaction between the host immune system and certain external factors, such as viral infections and gut microbiota. The most extensively studied TLRs in the setting of CD seem to be TLR2 and TLR4, but current evidence of their exact role in the pathogenesis is limited.^111^ TLRs have also been linked to the regulation of B cells in SLE, specifically TLR7 and TLR9; however, further investigations are warranted to understand the dynamics of this link.^112^ Finally, healthy controls with high titres of ANA, which are highly frequent in patients with SLE, showed upregulation of a gene that encodes for the CD transglutaminase autoantigen 2.^113^ Speculatively, this could be interpreted as an association between gliadin autoreactivity and incomplete forms of SLE.

Investigating the opposite relationship, a large Swedish population-based cohort study by Ludvigsson *et al*^10^ found a threefold increased risk of SLE in patients with Marsh stage III biopsy-verified CD compared with the general population. We found a weighted pooled prevalence of Marsh stage III biopsy-verified CD of 1.8%. Except from the opposite outcome, the study design and study population in our study are not directly comparable with the Swedish study. Moreover, when comparing our estimate of 1.8% with the CD prevalence in the general population of the four studies reporting Marsh stage III (population prevalence ranged between 0.4% and 1.0%), we found a twofold to fourfold increased CD prevalence in patients with SLE.

When interpreting our results, some methodological aspects should be taken into consideration. First, in our study, we required the diagnosis of CD to be biopsy-verified. Several patients with positive CD serological markers in studies included in the primary analysis declined or for other reasons did not undergo endoscopic examination with biopsy and were therefore classified as patients without CD. Second, we excluded studies in our analysis which included patients with a prior diagnosis of CD, and several of the included studies likewise excluded patients with a prior diagnosis of CD from their study populations. Third, testing for IgA deficiency was conducted in only 5 out of 14 studies. Previous studies showed that the presence of IgA deficiency reduces the sensitivity of serological screening for CD.^114^ This could potentially lead to undetected cases and underestimation of the true CD prevalence. Finally, several patients in the included studies were asymptomatic and were therefore less likely to test positive for CD serological markers or show villous atrophy on endoscopic examination with biopsy.

The threefold increased prevalence of SLE in patients previously diagnosed with biopsy-verified CD reported by Ludvigsson *et al*^10^ indicates that the temporal relationship between the two diseases could play a role. From a clinical perspective, gastrointestinal symptoms in a patient without SLE would be more likely to be interpreted as symptoms of a primary gastrointestinal disease, while the same symptoms in a patient with SLE would perhaps be interpreted as related to SLE itself or to SLE medication, thus less likely to lead to a diagnostic investigation of CD. This aspect may also influence the differences between our studies.

We also investigated the seroprevalence of CD markers in patients with SLE. The global seroprevalence of CD markers in the general population was reported in a meta-analysis to be 1.4%.^14^ Several studies have reported an increased seroprevalence in patients with autoimmune diseases.^94 96 107^ Our pooled estimate of 3.7% (among those with any positive TTG or EMA) is higher than the prevalence of biopsy-verified CD and markedly higher than the prevalence globally. However, our included studies showed a substantial heterogeneity (I^2^=74.2%). When excluding studies that reported a remarkably high seroprevalence (>20.0%), our pooled estimate was 3.1%. However, we noted a considerably higher pooled prevalence of CD (11.6%) when defining CD seropositivity as a positive serology of any of TTG, EMA and AGA. Common antibodies in SLE include ANA, which is present in nearly all patients diagnosed with SLE. This finding suggests that seropositivity with ANA might be correlated with a higher prevalence of seropositivity with AGA without positive serology for any of TTG or EMA. Given the lower specificity of AGA for CD, the association of seropositivity of solely AGA as a CD serological marker seems less supportive of an association of CD in patients with SLE.^23^ Altogether, our estimates of the seroprevalence of CD markers in patients with SLE seem higher than in the general population, although they have to be interpreted with caution due to a general increase in the prevalence of autoantibodies and the significant variability in range among patients with SLE.^115^

Publications included in our study differed substantially in methodology. Differences were seen in the classification of biopsy-verified CD, testing of CD serological markers and cut-off points for serological markers. Furthermore, the included publications cover an extensive period of time (2001–2021) during which various guidelines for detecting CD have been used and the availability of endoscopic examinations varied. Also, the diagnostic criteria for SLE have changed during this time period.^116^ These factors may have influenced our results. In addition, the study population varied in age, size, comorbidities, genetics, ethnicity and disease severity of SLE. Data on ongoing treatment for SLE were reported only in three studies included in the primary analysis. These study characteristics and disease-related aspects need to be accounted for when interpreting our results. To further investigate the heterogeneity between studies, we performed several meta-regression analyses but found no significant associations.

We also observed a difference in the prevalence of CD in patients with SLE by region, with the highest pooled weighted estimates for the studies from North Africa, followed by Asia, Europe and South America. It is well known that ethnicity is of importance not only in the development but also in the course and prognosis of an SLE disease, where patients from Africa, East Asia, South Asia and South America are more likely to develop a more severe disease.^117^ Although our findings might be explained by various study designs and selection of study populations in the different studies, one might speculate if the characteristics of the SLE population could affect the association between the two conditions, that is, whether a stronger association might be observed in patients with SLE with a more severe disease.

Currently, there are no leading gastroenterological societies that advocate routine screening of CD in patients with SLE. General guidelines from the American College of Gastroenterology propose screening for CD when individual clinical signs and symptoms are suggestive of the disease and recommends small bowel biopsies regardless of the outcome of serological tests among both adults and children.^118^ They acknowledge, however, that this case-by-case approach may not be sufficient to identify all patients with CD and that no consensus exists on the associated diseases that necessitate screening for CD. The European Society for Paediatric Gastroenterology Hepatology and Nutrition proposes testing for CD in certain cases, such as autoimmune conditions, listing type 1 diabetes mellitus, thyroid disease and autoimmune liver disease as examples, and without any explicit reference to SLE.^119^ Our findings of a CD prevalence comparable with that of the general population do not support standardised screening for CD in patients with SLE. Nonetheless, it is essential to acknowledge overlapping clinical manifestations of CD and SLE. Although CD is characterised by diarrhoea and abdominal pain, it may also present with various extraintestinal manifestations such as arthralgia, thus resembling rheumatological conditions, including SLE. Patients with SLE may likewise be afflicted by the aforementioned symptoms, with roughly half of them experiencing gastrointestinal symptoms.^93^ To what extent overlapping symptoms lead to a higher proportion of undiagnosed CD in patients with SLE is not known but could indeed be the case. Moreover, it may be that symptoms related to an underlying CD are interpreted as symptoms related to immunomodulating medications used in SLE and thus potentially mask or delay a diagnosis of CD, whereas the opposite seems less likely. Patients with SLE have a reduced health-related quality of life and a considerable disease burden with sometimes disabling symptoms.^6^ This overlap of symptoms, together with the disease burden of SLE, may suggest a need for increased vigilance among healthcare professionals of signs consistent with CD in patients with SLE and warrants a low threshold for CD diagnostic screening. However, we do not believe our results support the introduction of routine screening for CD in this patient population. Instead, screening with serological markers of CD followed by a small bowel biopsy in seropositive patients could be warranted when there is clinical suspicion of CD or in the presence of additional risk factors for CD. More studies with larger study populations from different regions are necessary to further investigate and develop modalities of potential diagnostic algorithms for CD in patients with SLE.

This meta-analysis has several strengths. First, four major databases (Medline, Embase, Cochrane and Web of Science Core Collection) were used to identify relevant studies, enabling a comprehensive screening of existing literature related to the research question. Second, the primary analysis was restricted to histologically confirmed cases of CD to increase internal validity since diagnosis of CD exclusively based on serological markers could overestimate the true prevalence. Third, we performed several meta-regression analyses to explore potential associations between study characteristics and CD prevalence in SLE that could shed light on our findings. Finally, we conducted a meta-analysis of serological markers of CD in patients with SLE to compare the results of our primary analysis of histologically confirmed CD.

We acknowledge some limitations to our study. First, we lacked data on CD in patients with SLE from large parts of the world, including East Asia, Oceania and Sub-Saharan Africa. Second, we did not have data on the ethnicity of the included patients on an individual level. Our results should therefore be inferred with caution to different ethnic populations. Third, the included studies did not allow analysis of cases without biopsy-verified CD. Fourth, our results might underestimate the true prevalence of CD since several study participants with elevated serological markers of CD did not undergo an endoscopic examination with biopsy and were therefore classified as patients without CD in our primary analysis. Finally, data on immunosuppressive treatment were scarce in the included studies and not accounted for in our analysis. This should be considered when interpreting the results.

## Conclusions

In conclusion, through a systematic review and meta-analysis, we found a prevalence of biopsy-verified CD in patients with SLE similar to the prevalence in the general population. Currently, no major gastroenterology or rheumatology society advocates routine screening of CD in patients with SLE. Our results do not support the implementation of routine screening in patients with SLE. However, given the risk of underestimation of the prevalence in this study and the clinical resemblance between the conditions, individual screening could be considered in patients with SLE in cases of clinical suspicion or additional risk factors for CD.

## Data Availability

All data relevant to the study are included in the article or uploaded as supplementary information.
