# Supplementary material for: Prevalence of coeliac disease in patients with systemic lupus erythematosus: a systematic review and meta-analysis
Source: Lupus Sci Med. 2024 Feb 13;11(1):e001106. doi: 10.1136/lupus-2023-001106 (PMC10868192; doi:10.1136/lupus-2023-001106)
Supplement: Supplementary data [file lupus-2023-001106supp001.pdf]

Supplementary material

This supplement contains additional tables and figures to the study by Adonis Sotoodeh, Madeleine N. Hoang, Karin Hellgren, and Anders Forss:

"Prevalence of coeliac disease in patients with systemic lupus erythematosus: a systematic review and meta-analysis"

Table of content

Table S1 Studies included in the meta-analysis on prevalence of serological markers of coeliac disease in patients with systemic lupus erythematosus (page 2)

Table S2 Quality assessment of included studies according to the Joanna Briggs critical appraisal tool (page 3)

Figure S1 PRISMA Flow Diagram and search strategies (pages 4-8)

Figure S2 Prevalence of coeliac disease restricted to cases of Marsh stage III in patients with systemic lupus erythematosus (page 9)

Figure S3 Prevalence of coeliac disease in patients with systemic lupus erythematosus stratified by region (page 10)

Figure S4 A-E Meta-regression analyses of coeliac disease in patients with systemic lupus erythematosus (pages 11-13)

Figure S5 Prevalence of coeliac disease antibodies (antitissue transglutaminase, antiendomysial antibodies, and antigliadin antibodies) in patients with systemic lupus erythematosus (page 14)

Table S1 Studies included in the meta-analysis on prevalence of serological markers of coeliac disease in patients with systemic lupus erythematosus.

**Study**	**Patients** n	**Female** (%)	**Age** mean (range)	**Antibodies**	**Seropositive (TTG/EMA)** n (%)	**Seropositive (TTG/EMA/AGA)** n (%)
**Author**, publication year						
Aikawa, 2012	41	85.4	14 (NA)	EMA	1 (2.4)	1 (2.4)
AlEnzi, 2020	115	90.4	27 (NA)	TTG/EMA/AGA	5 (4.3)	15 (13.0)
Ben Abdelghani, 2012	24	91.7	36 (18–52)	TTG/EMA/AGA	2 (8.3)	7 (29.2)
Caio, 2018	35	82.9	NA (NA)	TTG/EMA/AGA	1 (2.9)	3 (8.6)
Elhami, 2018	100	92.0	49 (16–76)	TTG	1 (1.0)	1 (1.0)
Gheita, 2012	10	80.0	12 (NA)	TTG	6 (60.0)	6 (60.0)
Mader, 2003	61	NA	NA (NA)	EMA/AGA	0 (0.0)	27 (44.3)
Marai, 2004	100	88.0	31 (15–56)	TTG/EMA	3 (3.0)	3 (3.0)
Picceli, 2013	194	92.8	39 (17–67)	TTG/EMA	11 (5.7)	11 (5.7)
Rensch, 2001	103	NA	NA (NA)	EMA/AGA	0 (0.0)	24 (23.3)
Sahin, 2019	50	88.0	15 (NA)	TTG/EMA	3 (6.0)	3 (6.0)
Shamseya, 2020	100	900	35 (19–55)	TTG/EMA	10 (10.0)	10 (10.0)
Soltani, 2021	130	81.5	32 (16–60)	TTG/EMA/AGA	5 (3.8)	6 (4.6)

Table S2 Quality assessment of included studies according to the Joanna Briggs Critical Appraisal Tool

Author, publication year	Assessment (yes/no) by question[a]									
	**Q1**	**Q2**	**Q3**	**Q4**	**Q5**	**Q6**	**Q7**	**Q8**	**Q9**	**Total number of yes**
Aikawa, 2012	Y	Y	N	Y	N	Y	Y	Y	N	6
AlEnzi, 2020	Y	Y	Y	N	Y	Y	Y	Y	Y	8
Ben Abdelghani, 2012	Y	Y	N	N	Y	Y	Y	Y	Y	7
Caio, 2018	Y	Y	N	N	Y	N	Y	Y	Y	7
Elhami, 2018	Y	Y	Y	Y	Y	Y	Y	Y	Y	9
Gheita, 2012	Y	Y	N	Y	Y	N	Y	Y	Y	7
Linzmeyer, 2020	Y	Y	Y	N	Y	Y	Y	Y	Y	8
Mader, 2003	Y	Y	N	N	Y	N	Y	Y	Y	6
Marai, 2004	Y	Y	Y	N	Y	Y	Y	Y	Y	8
Picceli, 2013	Y	Y	Y	Y	Y	Y	Y	Y	Y	9
Rensch, 2001	N	Y	Y	N	N	Y	Y	Y	N	5
Sahin, 2019	Y	Y	Y	Y	Y	Y	Y	Y	Y	9
Shamseya, 2020	Y	Y	Y	Y	Y	Y	Y	Y	Y	9
Soltani, 2021	Y	Y	Y	Y	Y	Y	Y	Y	Y	9

Y= Yes; N= No

[a] Munn Z, Moola S, Lisy K, Riitano D, Tufanaru C. Chapter 5: Systematic reviews of prevalence and incidence. In: Aromataris E, Munn Z (Editors). JBI Manual for Evidence Synthesis. JBI, 2020

BMJ Publishing Group Limited (BMJ) disclaims all liability and responsibility arising from any reliance placed on this supplemental material which has been supplied by the author(s)

PRISMA 2020 Flow Diagram

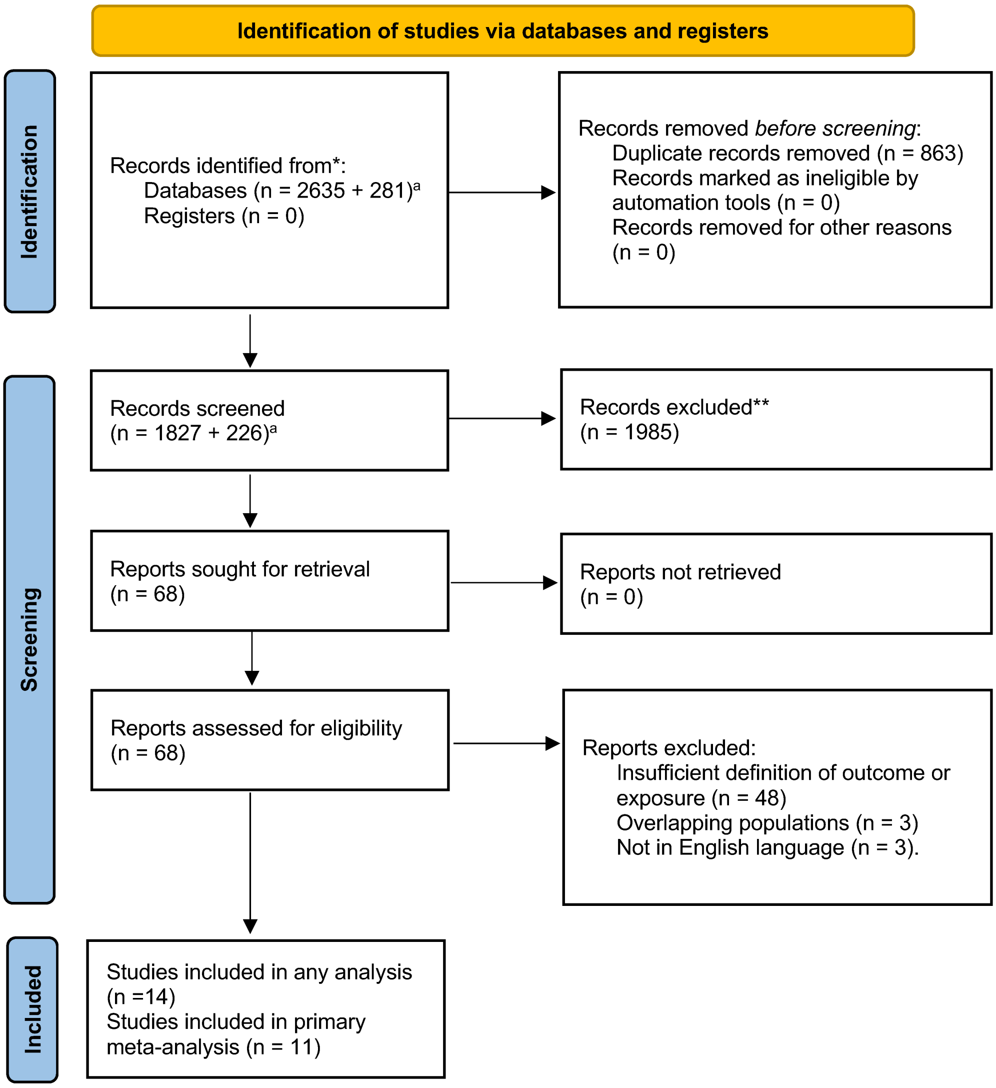

From: Page MJ, McKenzie JE, Bossuyt PM, Boutron I, Hoffmann TC, Mulrow CD, et al. The PRISMA 2020 statement: an updated guideline for reporting systematic reviews. BMJ 2021;372:n71. doi: 10.1136/bmj.n71

[a]The first search was conducted 13 June 2022. An updated search was conducted 9 July 2023. Identification of records is shown separately for each search (number of records for search 1 + number of records for search 2).

1. Medline

Interface: Ovid MEDLINE(R) and Epub Ahead of Print, In-Process & Other Non-Indexed Citations and Daily<br><br>Date of Search: 2022-06-13, updated search 2023-07-09<br><br>Number of hits: 461 + 36<br><br>Comment:	Field labels<br><br>• exp/ = exploded MeSH term<br>• / = non exploded MeSH term<br>• ti,ab,jw,kf. = title, abstract, journal word and author keywords<br>• adjx = within x words, regardless of order<br>• * = truncation of word for alternate endings

1. exp Lupus Erythematosus, Systemic/
2. exp Lupus Nephritis/
3. (SLE OR Systemic Lupus Erythematosus OR Lupus).ab,kf,jw,ti.
4. 1 OR 2 OR 3
5. Celiac Disease/
6. exp Glutens/
7. exp Transglutaminases/
8. (celiac* OR celiak* OR coeliac* OR coeliak* OR 'non-tropical sprue' OR 'nontropical sprue').ab,ti,kw
9. (Gluten* OR gliadin* OR wheat).ab,kf,ti.
10. (endomys* OR antiendomys* OR ema OR aea OR transglutamin* OR anti-transglutamin* OR trans glutamin* OR ttg OR tta OR antigliadin OR aga).ti,ab,kf.
11. ((villus or villous) adj3 atroph*).ti,ab,kf.
12. 5 OR 6 OR 7 OR 8 OR 9 OR 10 OR 11
13. 4 AND 12
14. limit 13 to yr="1990 - 2022"

*14 (updated search). limit 13 to dt=20220614-20230613

2. Embase

Interface: embase.com<br><br>Date of Search: 2022-06-13, updated search 2023-07-09<br><br>Number of hits: 1463 + 212<br><br>Comment: Emtree is the controlled vocabulary in Embase	Field labels<br><br>• /exp = exploded Emtree term<br>• /de = non exploded Emtree term<br>• ti,ab,kw = title, abstract and author keywords<br>• NEAR/x = within x words, regardless of order<br>• * = truncation of word for alternate endings

1. 'systemic lupus erythematosus'/exp OR 'systemic lupus erythematosus'
2. 'lupus erythematosus nephritis'/exp OR 'lupus erythematosus nephritis'
3. sle:ti,ab,kw OR lupus:ti,ab,kw
4. #1 OR #2 OR #3
5. 'celiac disease'/de
6. 'gluten'/de
7. 'gliadin'/de
8. 'protein glutamine gamma glutamyltransferase'/de
9. 'protein glutamine gamma glutamyltransferase 2'/de
10. 'endomysium antibody'
11. 'endomysium'
12. celiac*:ti,ab,kw OR celiak*:ti,ab,kw OR coeliac*:ti,ab,kw OR coeliak*:ti,ab,kw OR 'non-tropical sprue':ti,ab,kw OR 'nontropical sprue':ti,ab,kw
13. gluten*:ti,ab,kw OR gliadin*:ti,ab,kw
14. endomys*:ti,ab,kw OR antiendomys*:ti,ab,kw OR ema:ti,ab,kw OR aea:ti,ab,kw OR transglutamin*:ti,ab,kw OR 'anti transglutamin*':ti,ab,kw OR 'trans glutamin*':ti,ab,kw OR ttg:ti,ab,kw OR tga:ti,ab,kw OR 'antigliadin*':ti,ab,kw OR aga:ti,ab,kw
15. ((villus OR villous) NEAR/3 atroph*):ti,ab,kw
16. #5 OR #6 OR #7 OR #8 OR #9 OR #10 OR #11 OR #12 OR #13 OR #14 OR #15
17. #4 AND #16 AND [01-01-1990]/sd

3. Cochrane Library

Interface: Wiley<br><br>Date of Search: 2022-06-13, updated search 2023-07-09<br><br>Number of hits: 14 + 1	Field labels<br><br>• ti,ab,kw = title, abstract and author keywords<br>• NEAR/x = within x words, regardless of order<br>• * = truncation of word for alternate endings<br>• [ ] = MeSH descriptor, explode all trees

1. [Lupus Erythematosus, Systemic]
2. [Lupus Nephritis]
3. (SLE OR Systemic Lupus Erythematosus OR Lupus):ti,ab,kw
4. #1 OR #2 OR #3
5. [Celiac Disease]
6. [Glutens]
7. [Transglutaminases]
8. (celiac* OR celiak* OR coeliac* OR coeliak* OR 'non-tropical sprue' OR 'nontropical sprue'):ti,ab,kw
9. (gluten* OR gliadin*):ti,ab,kw
10. (endomys* OR antiendomys* OR ema OR aea OR transglutamin* OR anti-transglutamin* OR trans glutamin* OR ttg OR tta OR antigliadin OR aga):ti,ab,kw
11. ((villus or villous) NEAR/3 atroph*):ti,ab,kw
12. #5 OR #6 OR #7 OR #8 OR #9 OR #10 OR #11
13. #4 AND #12

with Cochrane Library publication date from Jan 1990 to June 2022, updated search until July 2023

4. Web of Science Core Collection

<table>
<tr><td>Interface: Clarivate Analytics

Date of Search: 2022-06-13, updated search 2023-07-09

Number of hits: 697 + 29</td><td>Field labels

• TS/Topic = title, abstract, author keywords and Keywords Plus
• NEAR/x = within x words, regardless of order
• * = truncation of word for alternate endings

Note: sometimes “quotation marks” are needed for single search terms to avoid automatic term mapping (lemmatization).</td></tr>
<tr><td colspan="2">1. TOPIC: ("Systemic Lupus Eryt*”)
2. TOPIC: ("Lupus Nephritis")
3. TOPIC: (SLE OR Lupus)
4. #1 OR #2 OR #3
5. TOPIC: ((celiac* OR celiak* OR coeliac* OR coeliak* OR “non-tropical sprue” OR “nontropical sprue”))
6. TOPIC: ((gluten* OR gliadin*))
7. TOPIC: ((endomys* OR antiendomys* OR ema OR aea OR transglutamin* OR anti-transglutamin* OR "trans glutamin*" OR ttg OR tta or antigliadin or aga))
8. TOPIC: (((villus OR villous) NEAR/3 atroph*))
9. #5 OR #6 OR #7 OR #8
10. #4 AND #9
Limited to publication date: 1990-01-01 – 2022-06-13, updated until 2023-07-09</td></tr>
</table>

Figure S1 PRISMA Flow Diagram and search strategies.

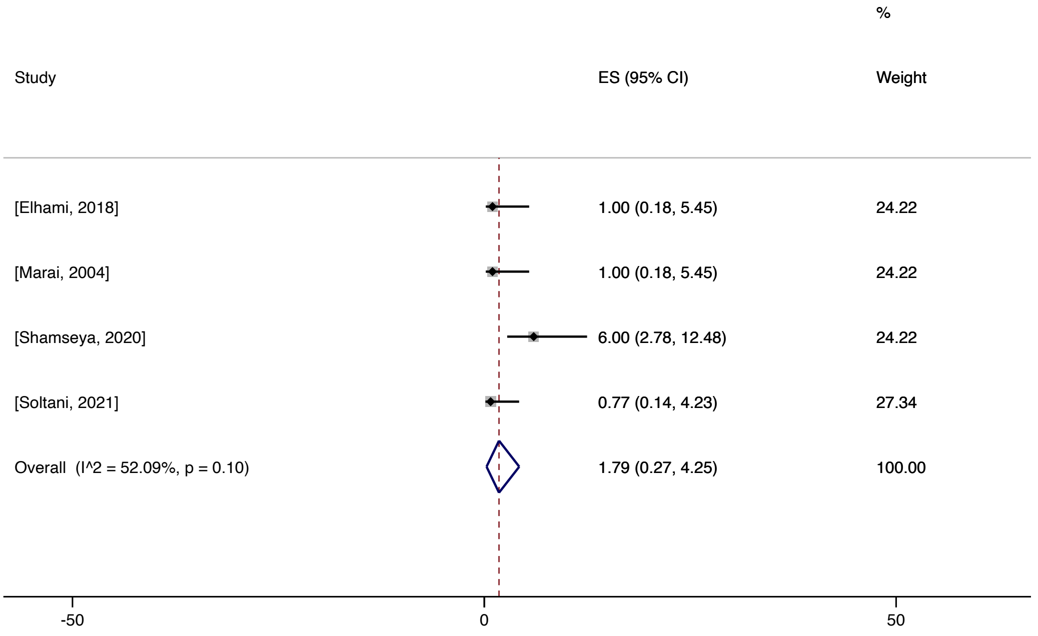

Figure S2 Prevalence of coeliac disease restricted to cases of Marsh stage III in patients with systemic lupus erythematosus.

CI, confidence interval; I^2, heterogeneity; p, p-value

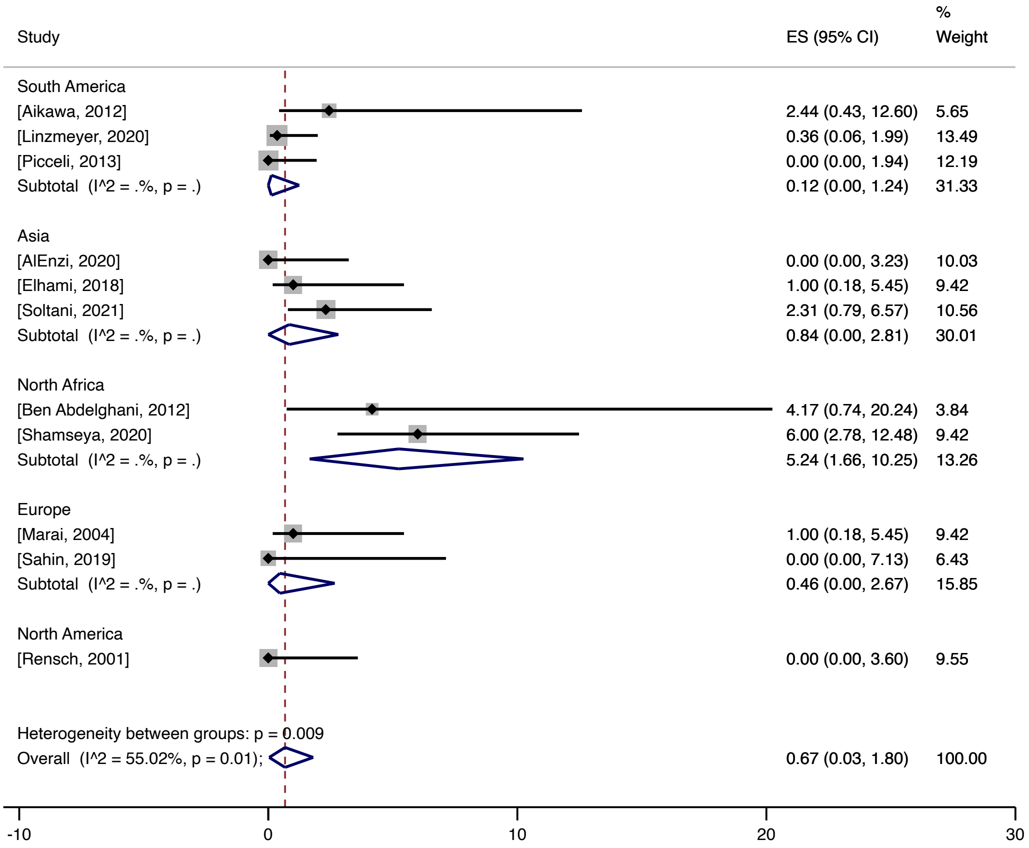

Figure S3 Prevalence of coeliac disease in patients with systemic lupus erythematosus stratified by region.

CI, confidence interval; I^2, heterogeneity; p, p-value

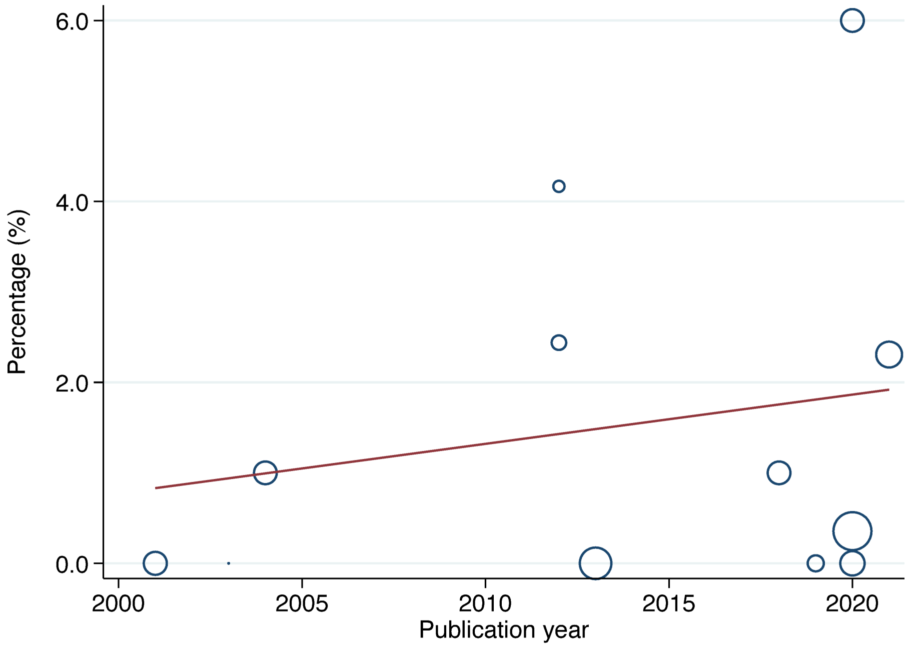
6.0
4.0
2.0
0.0
Percentage (%)
2000
2005
2010
2015
2020
Publication year

A)

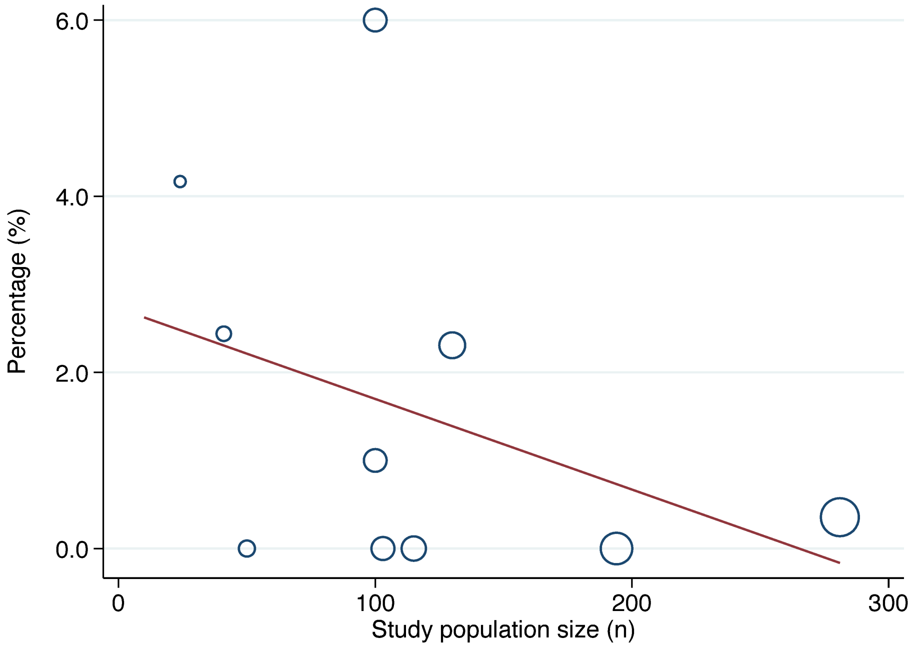
6.0
4.0
2.0
0.0
Percentage (%)
0
100
200
300
Study population size (n)

B)

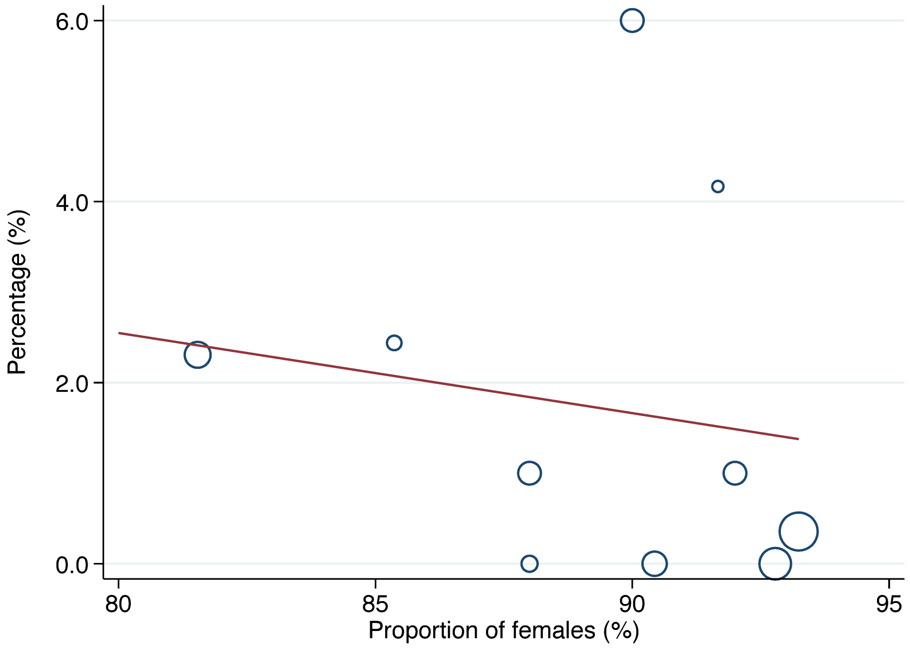
6.0
4.0
2.0
0.0
Percentage (%)
80
85
90
95
Proportion of females (%)

C)

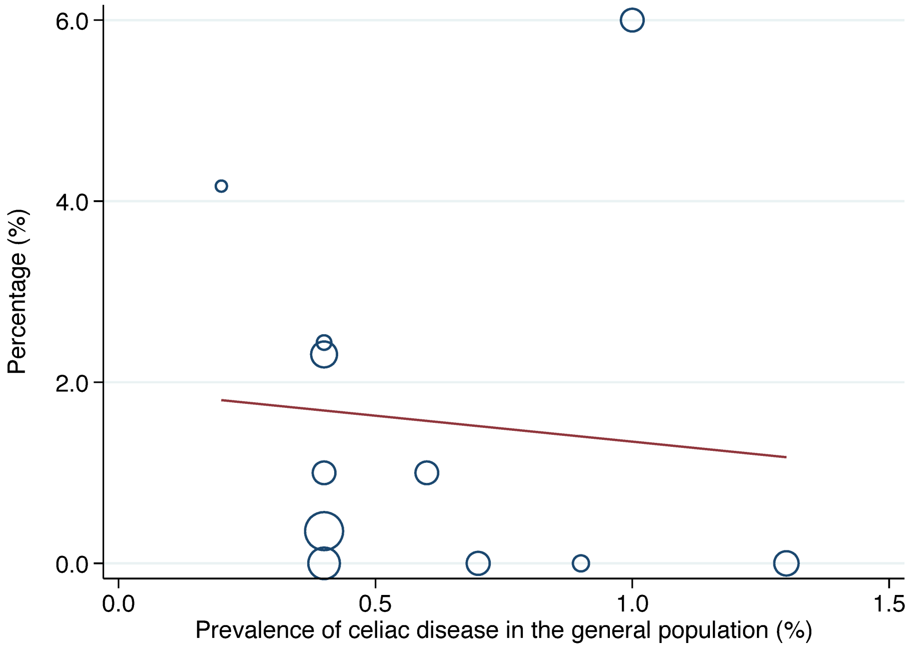
6.0
4.0
2.0
0.0
Percentage (%)
0.0
0.5
1.0
1.5
Prevalence of celiac disease in the general population (%)

D)

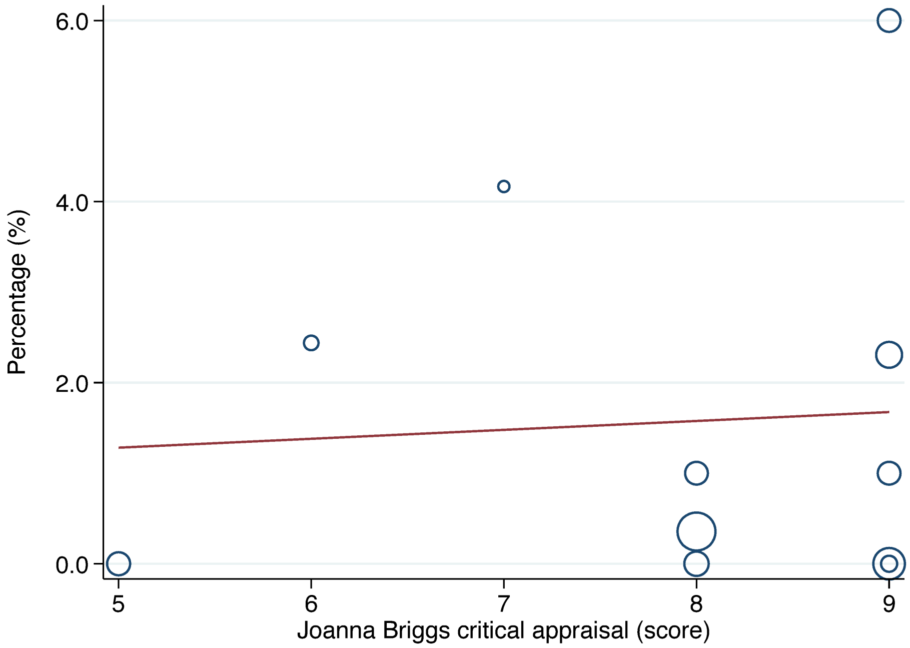

E)

Figure S4 A-E (A) Meta-regression of the association between publication year and prevalence of coeliac disease (CD) in patients with SLE. Y-axis shows percentage with CD. X-axis displays publication year for each study. (B) Meta-regression of the association between study population size and prevalence of CD in patients with SLE. Y-axis shows percentage with CD. X-axis displays the study population size for each study. (C) Meta-regression of the association between the proportion of females and the prevalence of CD in patients with SLE. Y-axis shows percentage with CD. X-axis displays the proportion of females in each study. (D) Meta-regression of the association between the prevalence of CD in the general population and the prevalence of CD in patients with SLE. Y-axis shows percentage with CD. X-axis displays the prevalence of CD in the general population where the study took place. (E) Meta-regression of the association between the Joanna Briggs critical appraisal score and the prevalence of CD in patients with SLE. Y-axis shows percentage with CD. X-axis displays the Joanna Briggs assessment scores for each study.

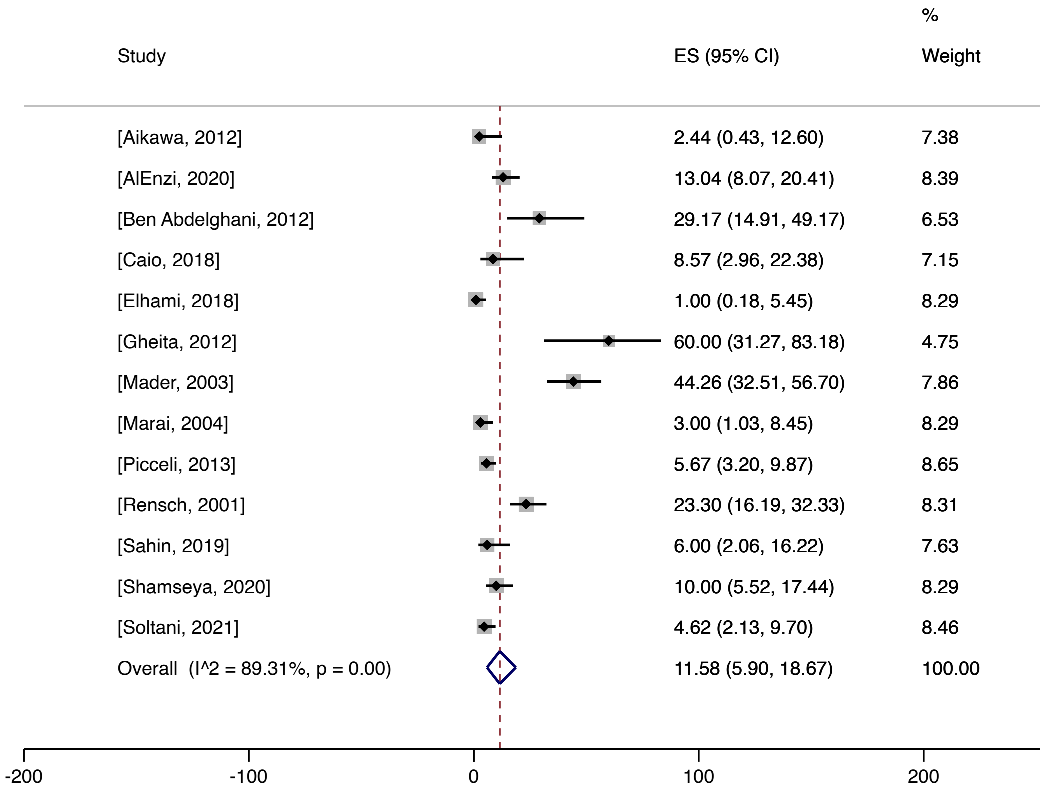

Figure S5 Prevalence of coeliac disease antibodies (antitissue transglutaminase, antiendomysial, and antigliadin antibodies) in patients with systemic lupus erythematosus.

CI, confidence interval; I^2, heterogeneity; p, p-value
